# Glaucoma drainage device surgery in children and adults: a comparative study of outcomes and complications

**DOI:** 10.1007/s00417-017-3584-2

**Published:** 2017-02-01

**Authors:** Achilleas Mandalos, Velota Sung

**Affiliations:** Birmingham and Midland Eye Centre, Sandwell and West Birmingham Hospitals NHS Trust, Dudley Road, Birmingham, B18 7QH UK

**Keywords:** Glaucoma drainage device, Children, Adults, Comparative outcomes, Complications

## Abstract

**Purpose:**

To compare the postoperative outcomes and complications of glaucoma drainage device (GDD) surgery in pediatric (<18 years old) and adult patients.

**Methods:**

Retrospective, comparative study including all patients who underwent Baervedlt or Molteno device surgery by the same surgeon. Success criteria included postoperative intraocular pressure (IOP) between 6 and 21 mmHg and a 20% reduction from baseline.

**Results:**

Fifty-two children (69 eyes) and 130 adults (145 eyes) were included. Mean IOP and number of medications were significantly reduced postoperatively in both groups. Overall failure rate was similar in children and adults. However, GDD failed earlier in adults than in children. Hypotony was the most common complication in both groups in the first 6 months postoperatively. Later on, bleb encapsulation was more frequent in children, while corneal decompensation tended to be more frequent and occurred earlier in adults. Children also had a higher rate of infectious endophthalmitis and required tube repositioning more frequently than adults.

**Conclusions:**

GDD surgery presents different postoperative challenges in children and adults, and the surgeon should remain vigilant for complications throughout the postoperative period, especially for signs of endophthalmitis or bleb encapsulation in pediatric patients. On the other hand, adults may be more prone to early corneal decompensation.

## Introduction

Glaucoma drainage devices (GDDs) have been developed as an additional option to trabeculectomy in the surgical management of refractory or complex glaucoma. Their use has been growing in popularity in recent years to also include cases of simple open-angle glaucoma. The Tube vs. Trabeculectomy Study, a randomized clinical trial on glaucoma patients with previous trabeculectomy and/or cataract surgery, showed a superior 5-year success rate for GDD compared to augmented trabeculectomy with a lower reoperation rate and a similar safety profile [1, 2].

GDDs are used in the surgical management of adult as well as pediatric glaucoma. Compared with adults, children have smaller eyes with highly elastic scleral tissue, which frequently makes implantation of the GDD challenging. In addition, differences in the type of glaucoma between children and adults may affect the surgical outcome of GDD, as primary congenital and developmental glaucoma predominate in children, whereas chronic open-angle glaucoma is the predominant type of glaucoma in adults.

Although there are several reports in the literature on the safety and efficacy of various types of GDD implantation in children and adults, there is no comparative study on the success and complications of GDD between these age groups. We reported in our previous study on the long-term outcomes and complications of GDD surgery in children in comparison to the existing literature [3]. The purpose of this study was to compare the outcomes and complications of GDD surgery in our pediatric series with adult patients coming from the same population who were operated upon by the same surgeon at a tertiary glaucoma referral center.

## Methods

This was a retrospective, comparative study including all glaucoma patients who underwent GDD surgery by the same surgeon (V.S.) at Birmingham and Midland Eye Centre during the period from January 1, 2004 to December 31, 2011. Data were retrieved from the case notes. The parameters recorded included best corrected visual acuity (BCVA), intraocular pressure (IOP) and antiglaucoma medications at the last preoperative clinic visit and at 3, 6 and 12 months postoperatively and every 6 months thereafter. Any complications and further procedures throughout the follow-up period were also recorded. Data collection was censored at the last clinic visit or at the time of failure. For patients who were lost to follow-up, data collection was censored at their last clinic visit. The study was approved by the local Research Ethics Committee (reference: 12/WM/0158).

The most common GDD implanted was the Baerveldt implant (surface area: 250 mm^2^ or 350 mm^2^) and less commonly the Molteno single-plate device (Table 1). The choice of GDD was determined by the surgeon after careful evaluation of the risk of postoperative hypotony, taking into account the type of glaucoma and any previous surgical or laser interventions as well as the accessibility of the sub-Tenon’s space for placement of the plate. The surgical technique was described in a previous report [3]. In brief, the conjunctiva and Tenon’s fascia were dissected in the superior temporal quadrant. Mitomycin C (MMC; 0.2–0.4 mg/ml) was applied on the equatorial sclera under the sub-Tenon pocket for 3–5 min. The plate was placed 8–10 mm posterior to the limbus and secured on the sclera with 7–0 polypropylene sutures. A 3–0 polyamide (Supramid, S. Jackson Inc., Alexandria, VA, USA) stent suture was introduced in the tube to avoid over-drainage. The tube was trimmed in a bevel-up fashion so that it would extend 1–2 mm beyond the limbus and was inserted into the anterior chamber through a scleral track made with a 25-gauge needle 2 mm posterior to the limbus. The tube was secured on the sclera with interrupted 9–0 nylon sutures and was ligated with a 6–0 polyglactin (dissolvable) suture. A 0.5-mm-long venting slit was made along the tube in all cases to allow for a small amount of leakage until the ligation suture dissolved, normally in about 6 week. Ethanol-preserved donor sclera was used to cover the tube near the limbus and was secured on host sclera with interrupted 9–0 nylon sutures. Tenon’s fascia and conjunctiva were closed with running 8–0 polyglactin sutures. Antibiotics and steroids were given intracamerally along with a supplemental orbital floor injection of a 50:50 mixture of 40 mg triamcinolone with 0.5% bupivacaine. The postoperative regimen included topical antibiotics and a tapering course of topical corticosteroid for 3 months. In addition, a 3-week tapering course of oral prednisolone (a commencing dose of 0.5 mg/kg) and a non-steroidal anti-inflammatory agent, e.g., ibuprofen, were routinely prescribed for patients aged 5 years or more.

**Table 1:** Baseline characteristics of patients

	Pediatric group		Adult group		*P* value
	N	%	N	%	
Age (years)					
Mean (SD)	8.3 (5.1)		50.3 (16.5)		<0.001*
Sex					0.184
Male	26	50.0	80	61.5	
Female	26	50.0	50	38.5	
Ethnicity					0.140
Caucasian	30	57.7	86	66.2	
Non-Caucasian	22	42.3	44	33.8	
Eye					0.618
Right	32	46.4	62	42.8	
Left	37	53.6	83	57.2	
Type of glaucoma					<0.001*
OAG	0	0.0	35	26.9	
Congenital	10	19.2	5	3.8	
ACG	0	0.0	8	6.2	
Uveitic	11	21.2	31	23.8	
Aphakic	16	30.8	15	11.5	
Ant. segment dysgenesis/aniridic	8	15.4	2	1.5	
Other^a^	7	13.5	34	26.2	
Family history of glaucoma					0.534
Yes	13	25.0	27	20.8	
No/unknown	39	75.0	103	79.2	
Previous glaucoma surgery					0.810
Yes	34	49.2	74	51.0	
No	35	50.8	71	49.0	
Lens status					0.188
Phakic	39	56.5	68	46.9	
Non-phakic (pseudophakic or aphakic)	30	43.5	77	53.1	
GDD type					0.08
Molteno	16	23.2	20	13.8	
Baerveldt (250 mm^2^ or 350 mm^2^ model)	53	76.8	125	86.2	
MMC use					0.589
Yes	60	86.9	122	84.1	
No	9	13.1	23	15.9	
Preoperative IOP (mmHg)					
Mean (SD)	29.4 (8.9)		28.1 (8.8)		0.097
No. of preop. antiglaucoma medications					
Mean (SD)	3.8 (1.2)		3.6 (1.1)		0.177
Preoperative VA (logMAR)					
Mean (SD)	0.79 (0.60)		0.70 (0.75)		0.062

*OAG*: open-angle glaucoma (includes primary OAG, pseudoexfoliative, pigmentary

juvenile OAG). *ACG*: angle-closure glaucoma (primary or secondary)

^a^Includes: mixed mechanism, phacomatose-related, neovascular, traumatic, secondary

to keratoplasty/retinal detachment surgery, etc.

*Denotes statistical significance

Mann–Whitney test, chi-square or Fisher’s exact test were used where appropriate

Success was defined as postoperative IOP from 6 to 21 mmHg and a reduction in IOP of at least 20% from preoperative levels without (complete success) or with (qualified success) topical and/or oral antiglaucoma medications. Additional criteria included no devastating vision loss (i.e., loss of light perception) and no need for further glaucoma surgery including cyclodestructive procedures. However, interventions to improve function of the GDD (such as removal of stent sutures, bleb needling and repositioning or shortening of the tube) were allowed. Hypotony was defined as IOP < 6 mmHg in at least two consecutive clinic visits or in the presence of hypotony complications (e.g., shallow anterior chamber and/or choroidal detachment). Bleb encapsulation was identified in the case notes as the description of a thick fibrous capsule around the plate restricting aqueous outflow and resulting in an IOP increase compared to that observed at the previous visit. Corneal decompensation was defined as persistent localized or generalized corneal edema in at least two consecutive visits. Endophthalmitis was defined as an inflammatory reaction involving both the anterior segment and the vitreal cavity.

Statistical analysis was performed using SPSS v16.0 (SPSS Inc., Chicago, IL, USA), and a *P* value of 0.05 was used to denote statistical significance. As data were not found to be normally distributed, non-parametric tests were used, namely the Mann–Whitney test for numerical variables, the chi-square test or Fisher’s exact test for frequencies and the Wilcoxon signed-ranks test for paired variables. Comparative survival analysis was performed using the Kaplan–Meier log-rank test.

## Results

Fifty-two children (69 eyes) aged less than 18 years at the time of GDD surgery and 130 adults (145 eyes) were included in the study. Mean age at surgery was 8.3 ± 5.1 years old for pediatric patients and 50.3 ± 16.5 years old for adult patients. The demographic and preoperative characteristics of the two age groups are shown comparatively in Table 1.

Patients were mostly Caucasian in both groups; however, South Asian ethnicity was more frequent in pediatric patients and Afro-Caribbean ethnicity was more prevalent in adults. As was expected, type of glaucoma differed between the groups, congenital-developmental and aphakic glaucoma being more frequent in children as opposed to chronic open-angle glaucoma and glaucoma from other causes, which prevailed in the adult group. Most patients were phakic at the time of surgery; however, most of the non-phakic children were aphakic, whereas most of the non-phakic adults were pseudophakic. No statistically significant differences were noted with regard to preoperative IOP, BCVA or the number of antiglaucoma medications. Approximately half of the patients in each group had undergone at least one glaucoma procedure prior to GDD surgery. Previous glaucoma surgeries included goniotomy, trabeculotomy, trabeculectomy, GDD implantation and cyclophotocoagulation. The previous surgery profile was slightly different between children and adults, although trabeculectomy was the most prevalent surgical procedure in both groups. No patient in the adult group had undergone goniotomy or trabeculotomy.

The Baerveldt implant was the most common GDD in both groups (Table 1). However, most of the children received the implant with a surface area of 250 mm^2^ (38 out of 69 eyes), whereas most of the adults had the 350-mm^2^ implant (92 out of 145 eyes).

The mean ± SD length of follow-up was 45.7 ± 25.2 months for pediatric patients and 32.6 ± 22.2 months for adults (*P* < 0.001, Mann–Whitney test). At the last follow-up or pre-censor visit, mean ± SD IOP was 15.5 ± 8.4 mmHg in children and 14.9 ± 6.7 mmHg in adults, whereas the mean ± SD number of antiglaucoma medications was 0.7 ± 1.1 and 0.4 ± 0.9, respectively. Mean IOP and mean number of medications were significantly reduced from baseline in both age groups (*P* < 0.001, Wilcoxon signed-ranks test) at all time-points. Moreover, IOP was similar between age groups up to 7 years postoperatively; the same was true for the number of medications.

Final postoperative mean ± SD BCVA was 0.74 ± 0.59 logMAR in pediatric patients and was not significantly different from preoperative levels, whereas in adults, it was 0.93 ± 0.93 logMAR and was significantly worse than preoperative measurements (*P* < 0.001, Wilcoxon signed-ranks test).

**Fig. 1:**
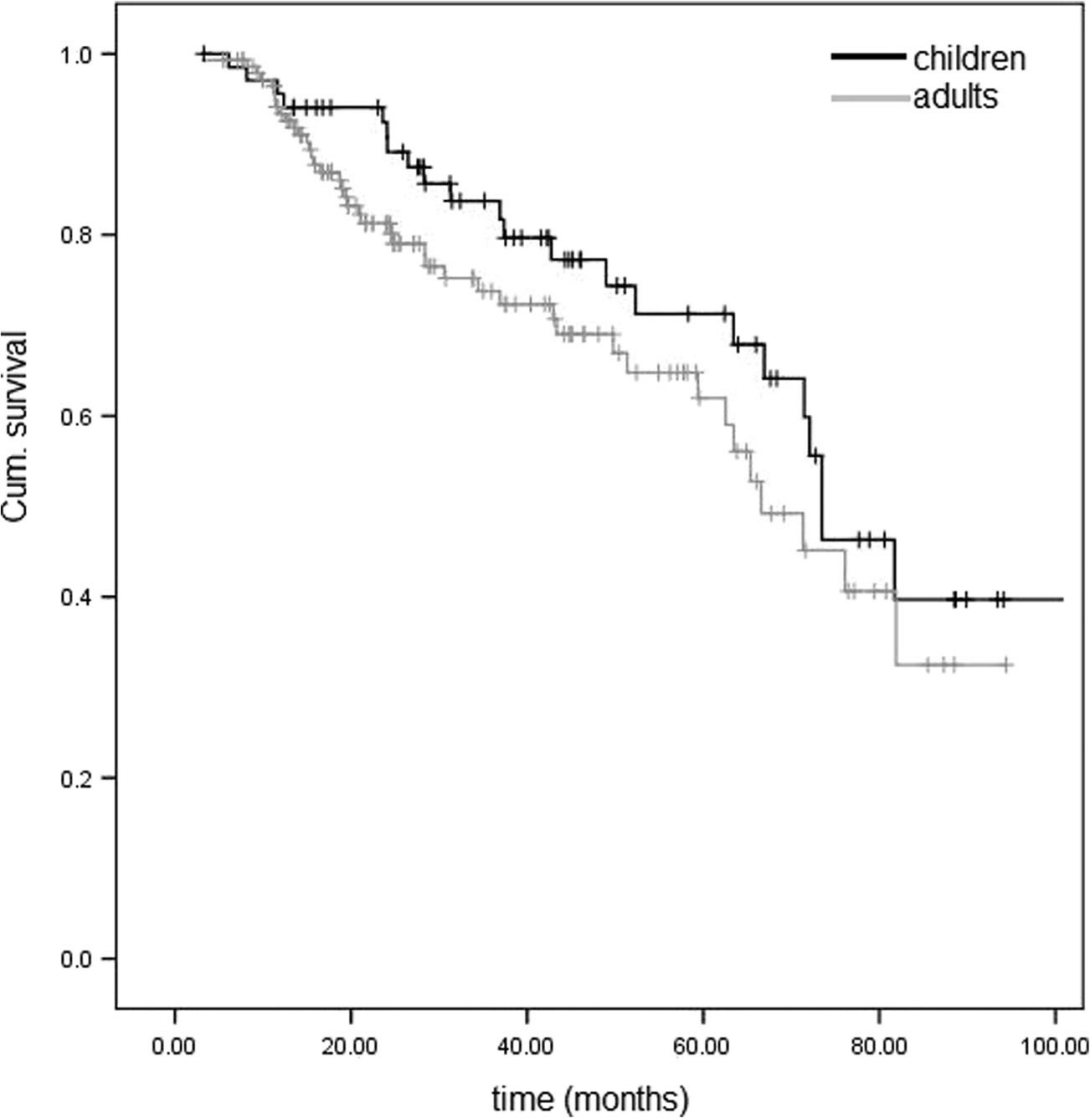
Kaplan–Meier survival curve for qualified success for pediatric and adult patients

The cumulative probabilities of complete and qualified success did not differ significantly between age groups (Table 2). Survival of GDD was also plotted on Kaplan–Meier curves (Fig. 1, showing curves for qualified success).

**Table 2:** Cumulative probabilities of GDD survival

	Complete success (cum. probability, %)		Qualified success (cum. probability, %)		No. of eyes remaining	
	Children	Adults	Children	Adults	Children	Adults
1 year	95.6	91.4	95.6	93.4	65	123
2 years	92.5	77.3	92.5	81.3	56	77
3 years	78.7	65.6	83.8	73.8	41	51
4 years	64.2	59.7	77.3	69.0	27	35
5 years	59.2	49.9	71.3	62.0	22	21
6 years	41.7	31.0	59.9	45.1	14	10
7 years	24.2	22.3	39.7	32.5	6	4
Survival log-rank test (*P* value)	0.252	0.185

Throughout the follow-up period, 23 (33.3%) pediatric and 42 (28.9%) adult cases were classified as failures. The distribution of reasons for GDD failure was marginally different between children and adults (Table 3). Four patients in each group had their GDD removed. In the pediatric group, all cases were associated with endophthalmitis. In adults, removal of the GDD was due to corneal decompensation in one case, phthisis in one case and enucleation of the eye for reasons unrelated to GDD in the other two cases.

**Table 3:** Reasons for failure for each age group

	Paediatric group		Adult group		*P* value
	N	%	N	%	
Reasons for failure					0.043*
IOP > 21 mmHg	6	26.1	5	11.9	
IOP < 6 mmHg	3	13.0	1	2.4	
<20% IOP reduction	5	21.8	17	40.5	
Further glaucoma surgery	1	4.3	1	2.4	
Cyclodestructive procedures	4	17.4	6	14.3	
Loss of light perception	0	0.0	8	19.0	
Other (e.g., GDD removal, enucleation)	4	17.4	4	9.5	
Total	23	100.0	42	100.0	

*Denotes statistical significance (Fisher’s exact test)

Although failure rates were similar between groups, median time to establishment of failure was significantly longer in children (37.3 months) than in adults (20.3 months), thus denoting that GDD may fail earlier in adults (*P* = 0.032, Mann–Whitney test).

In the first 6 months postoperatively, hypotony was the most frequent complication in both groups. After 6 months, cataract and bleb encapsulation were the most prevalent complications in the pediatric group, whereas corneal decompensation prevailed in adults (Table 4).

**Table 4:** Postoperative complications and procedures for each age group

	Pediatric group		Adult group		*P* value
	N	%	N	%	
Complications (within first 6 months)					
Hypotony	27	39.1	53	36.6	0.716
Choroidal effusion/detachment	15	21.7	39	26.9	0.417
Uveitis	6	8.7	13	9.0	0.948
Bleb encapsulation	1	1.4	1	0.7	0.542
Tube retraction	0	0.0	1	0.7	1.000
Corneal decompensation	0	0.0	4	2.8	0.308
Endophthalmitis	2	2.9	0	0.0	0.103
Retinal detachment	1	1.4	1	0.7	0.542
Cataract^a^	8	20.5	10	14.7	0.440
Postoperative procedures (within first 6 months)					
Intracameral injection of viscoelastic	27	39.1	48	33.1	0.388
Tying tube off	4	5.8	20	13.8	0.083
Removal/adjustment of stent suture	29	42.0	38	26.2	0.020*
Tube shortening/repositioning	2	2.9	4	2.8	1.000
Bleb needling or bleb revision	1	1.4	1	0.7	0.542
Lensectomy^a^	3	7.7	2	2.9	0.352
Complications (later than 6 months)					
Hypotony	9	13.4	14	9.7	0.421
Choroidal effusion/detachment	4	6.0	7	4.9	0.746
Uveitis	8	11.9	13	9.0	0.511
Bleb encapsulation	11	16.4	4	2.8	0.001*
Tube retraction	3	4.5	1	0.7	0.095
Corneal decompensation	8	11.9	22	15.3	0.518
Endophthalmitis	2	3.0	2	1.4	0.593
Retinal detachment	3	4.5	4	2.8	0.682
Cataract^a^	8	22.9	8	11.9	0.150
Postoperative procedures (later than 6 months)					
Intracameral injection of viscoelastic	3	4.5	15	10.4	0.151
Tying tube off	7	10.4	8	5.6	0.250
Removal/adjustment of stent suture	21	31.3	49	34.0	0.700
Tube shortening/repositioning	21	31.3	15	10.4	<0.001*
Bleb needling or bleb revision	10	14.9	5	3.5	0.007*
Lensectomy^a^	14	40.0	21	31.3	0.382

^a^Only phakic patients included

Chi-square or Fisher’s exact test were used where appropriate

*Denotes statistical significance

Compared with adults, pediatric patients exhibited more frequent bleb encapsulation during the late postoperative period (*P* = 0.001). Therefore, children also required bleb needling or bleb revision more frequently than adults (*P* = 0.007), although in one adult case, bleb revision was performed in order to excise a retention cyst. Furthermore, significantly more pediatric patients required tube repositioning or shortening procedures to correct the intraocular position of the tube, especially in cases where the tube progressively approximated or actually touched the corneal endothelium (*P* < 0.001).

Four pediatric patients developed endophthalmitis as opposed to two adult patients (*P* = 0.08). In two out of four pediatric cases of endophthalmitis, culture yielded positive results. The other two cases, although culture-negative, exhibited highly suspicious clinical signs of infective endophthalmitis, including hypopyon and vitritis. In contrast, both adult cases were sterile.

A higher proportion of adults developed corneal decompensation during follow-up, but this difference did not reach statistical significance (Table 4). However, the cornea seemed to decompensate earlier in adults as compared to children (median time of 18 months in adults versus 48.5 months in children, *P * = 0.004, Mann–Whitney test).

Although cataracts developed in a higher proportion of pediatric patients than adults, this difference did not reach statistical significance. Median (range) time to cataract development was 9 (3–54) months in children and 6 (2–30) months in adults (*P * = 0.56, Mann–Whitney test).

Hypotony was slightly more frequent in children, but this difference was not statistically significant. In a significant minority of cases (22.2% in children and 31.3% in adults), hypotony occurred after manipulation (adjustment or removal) of the stent suture or laser lysis of the ligating suture (and in one case, after bleb needling) due to inadequate IOP control. Hypotony was managed with the intracameral injection of a viscoelastic device and by ligation of the tube in refractory cases. The mean (SD) number of intracameral injections was 1.8 (1.0) injections in children and 2.6 (1.8) injections in adults and was marginally different between groups (*P * = 0.05, Mann–Whitney test). This difference in the need for intracameral injection of viscoelastic reflects the fact that hypotony settled more easily in children, whereas in adults it was more resistant to management. This may also explain the trend for more frequent tube tying in the adult group, especially in the first 6 months postoperatively (Table 4). It could be that children had healthier ciliary bodies and aqueous production and, therefore, were able to recover from hypotony more efficiently than adults. In fact, 30.3% of adult eyes had undergone cyclodestructive procedures prior to GDD surgery as opposed to only 18.8% in children and this may have resulted in a relatively lower capacity of adult eyes to recover from hypotony via aqueous production.

In the first 6 months postoperatively, significantly more pediatric patients required adjustment or removal of the stent suture to improve IOP control (Table 4). However, the overall rate of stent suture removal or adjustment throughout the follow-up period was similar between age groups, and no significant difference was noted in the median time of stent suture manipulation (5 months in children, 7 months in adults, *P * = 0.52, Mann–Whitney test). Due to the fact that the median time of stent suture manipulation in adults was shortly after the cut-off time-point used to separate early from late interventions, we also compared the rates of stent suture adjustment or removal in the first 12 months. The difference turned out to be non-significant (53.6% of children vs. 42.7% of adults, *P * = 0.14).

Finally, no significant association was found between the intraoperative use of MMC and late hypotony, bleb encapsulation, stent suture manipulation or endophthalmitis in either group (Fisher’s exact test). Similarly, IOP at 3 and 5 years postoperatively did not differ significantly between patients who had undergone augmented versus non-augmented surgery in either group (Mann–Whitney test).

## Discussion

In this study, overall GDD outcomes were similar between children and adults; however, there was a trend of GDD to fail earlier in adults, as indicated by the steeper survival curve in this group (Fig. 1). Although the reasons for this are not clear, it could be that the differences in the baseline characteristics of the two age groups affected the outcome. For example, the observed differences in the distribution of ethnicities in the two groups, combined with the inherent differences in the prevailing types of glaucoma and the slightly different previous surgical profile, may have had an impact in the natural history of GDD surgery in this study. Another potential explanation for the earlier failure of GDD in adults could be the longer duration of glaucoma medication use in this group; long-term topical therapy has been associated with an increased pro-inflammatory load in the conjunctiva and has been identified as a risk factor for failure of glaucoma filtration surgery. [4] Our success rates fell within a wide range of so-far published outcomes of GDD surgery. In the literature, success rates of 58.8–94.7% in children [5–13] and 64.0–91.7% in adults have been reported [1, 14, 15], thus indicating that outcomes may vary widely depending on the population studied and the definition of success. It is noteworthy that in our study, we used the IOPs at the last preoperative clinic visit as our baseline IOPs, and this may have underestimated the average IOPs before surgery, as most patients were on heavy antiglaucoma medications, particularly oral acetazolamide in the adult group as we tend to avoid oral acetazolamide in pediatric group because of the higher risk of potential side effects in children. This could potentially have skewed our outcomes towards failure due to failing to meet the 20% IOP reduction criterion. Indeed, this was the most frequent reason for classification as failure in the adult group (Table 3). In fact, if we allowed for an IOP reduction of less than 20% from baseline, the overall success rate at the last visit would have been as high as 82.1% in adults (67.6% complete and 14.5% qualified success) and 73.9% in children (55.1% complete and 18.8% qualified success).

Although no significant differences in the success rates were found between the two groups, one needs to consider the possibility of the GDD type affecting the outcome. Actually, in an ad-hoc analysis, GDD type seemed to influence the success/failure rate in the pediatric group (significantly higher rate of failure with the Molteno as compared to either type of the Baerveldt tube, although the sample was small and, therefore, of limited statistical power), whereas it did not seem to affect the failure rates in adults. The distribution of the three GDD types used in this study differed in the two groups and GDD selection was the result of careful preoperative evaluation and consideration of individual circumstances. In fact, the choice of GDD type very much depended on the size of the eye and risk factors for hypotony based on the surgeon’s experience and preoperative assessment. In younger children, the smaller eye size and the relatively smaller orbital volume would make implanting a 350-mm^2^ plate significantly more difficult; hence, a GDD with a smaller plate like the Baerveldt-250 or Molteno single plate would be technically more suitable. Furthermore, in eyes with a prior history of multiple cyclodestructive procedures or chronic uveitis, the risk of hypotony in the postoperative period is higher; therefore, a small plate size would be safer and more desirable.

Children had a significantly higher rate of bleb encapsulation than adults. This may denote a more aggressive scarring response of pediatric eyes, despite the intraoperative use of MMC. Previous studies reported a wide range of bleb encapsulation rates of 3.2–14.3% in children [5, 6, 16] and 2.0–27.1% in adults [2, 17]. These discrepancies may be attributed to variation in the definition of bleb encapsulation and differences in patient characteristics. Unlike trabeculectomy, it is our experience that bleb needling after GDD surgery does not offer sustainable improvement in IOP control; instead, it may cause acute hypotony complications and then exacerbation of bleb encapsulation that increases the risks of failure. In fact, 7 out of 11 children and 3 out of 6 adults that underwent bleb needling or bleb revision were ultimately classified as failures.

In our study, about 6% of pediatric patients suffered either definite or strongly suspected infectious endophthalmitis and had their GDD removed. In our previous study [3], we discussed this high incidence of endophthalmitis in relation to the published literature. In contrast, no adult patient developed infectious endophthalmitis, although there were two cases of sterile endophthalmitis. This discrepancy indicates that pediatric eyes may be more prone to infection and also reflects the inherent difficulties in postoperative management of children, including their relatively poor adherence to postoperative treatment and periocular hygiene rules. In addition, frequent eye rubbing in children may predispose these patients to conjunctival dehiscence and tube exposure, which are known risk factors for infection [18]. Conjunctival dehiscence shortly after retinal detachment surgery may have actually been the cause of endophthalmitis in one of our pediatric cases. Careful and meticulous closure of the conjunctiva and strict adherence to postoperative instructions are deemed important to reduce the risk of infection after GDD surgery.

Although antimetabolites reduce scar tissue formation, no association was found between the use of MMC and bleb encapsulation in this study. Furthermore, MMC was not found to be associated with endophthalmitis in either age group. However, these findings should be interpreted with caution due to the limited statistical power of our study and the rather small number of patients undergoing non-augmented GDD surgery.

Compared with adults, eye size in children can change more significantly as a result of changes in eye pressure due to the more elastic scleral tissue. Thus, any sustained increase (as in uncontrolled glaucoma) or decrease (after GDD surgery) in eye pressure can cause more significant changes in the size of the pediatric eye; hence, tube position may change more significantly in children. Moreover, the natural increase in eye size as part of children’s growth may result in tube retraction or a progressive change in the position of the intraocular part of the tube relative to the cornea. This may explain the observation that significantly more pediatric patients than adults required tube shortening or repositioning in our study.

Although not statistically significant, adults developed corneal decompensation more frequently than children. There is no direct comparison of corneal decompensation rates in adults and children in the available literature; however, rates of persistent corneal edema of up to 16% in adults [2] and up to 25% in pediatric patients undergoing GDD surgery have been reported [12]. Moreover, in our study, the cornea decompensated earlier in the adult group, which may be attributed to the fact that adult corneas may have fewer endothelial cells than children and, thus, may be more vulnerable to tube-induced endothelial cell loss. Although this hypothesis cannot be corroborated by specular microscopy data due to the retrospective nature of this study, the authors feel that it is advisable that the surgeon ensures proper positioning of the tube away from the corneal endothelium to minimise the risk of endothelial cell damage, and, in fact, we observed improvement in localized corneal edema after trimming or repositioning of tubes in certain cases. Additionally, regularly checking the mobility of the intraocular portion of the tube via pressure through the upper eyelid would help detect those patients at risk of tube-cornea touch who would benefit from further prophylactic tube shortening or repositioning. These measures also count for children, especially those with corneal changes (Haab’s striae) as a result of congenital glaucoma or chronic uveitis, who may also be at higher risk for corneal decompensation. Another potential reason for the relatively higher and quicker decompensation rate in adults could be the longer duration of the glaucoma condition suffered by adults and also the longer duration of glaucoma medication used in this group that may have had a negative impact on the health of corneal endothelium.

No proper cause for the slightly higher rate of cataract formation in the pediatric group was identified. However, it could be hypothesized that the more uncontrolled rubbing of the eyes in this group may have caused frequent impact of the tube against the anterior surface of the iris and the lens. Furthermore, children might be more susceptible to the development of lens opacities secondary to the long duration of topical steroid use postoperatively.

Our findings should be evaluated under consideration of the various limitations of the study. This was a retrospective, non-randomized study with variable follow-up length, and data retrieval was based on case notes; therefore, documentation bias cannot be excluded. Furthermore, post-hoc power analysis showed that the sample size was not sufficiently large to detect differences in the success/failure rates of GDD.

Despite these limitations, this is the first study directly comparing GDD outcomes and complications in children versus adults. The results presented here provide useful insight into potentially different challenges in postoperative care. Furthermore, patients came from the same population and were operated upon by the same surgeon, thus eliminating any surgeon and patient selection bias. Ideally, a prospective and adequately powered study would clarify whether GDD surgery carries a different prognosis in pediatric versus adult patients and whether the observed postoperative disparities reflect inherent differences in the surgical management of their glaucoma.

In summary, this comparative study showed that GDD surgery may have similar outcomes but presents different postoperative challenges in children and adults. This may be related to the inherent differences in the nature of glaucoma and eye characteristics between these groups. Hypotony seems to be common in both age groups in the first 6 months postoperatively but can be managed successfully in the majority of cases. However, in the late postoperative period, children may need bleb needling and tube repositioning more frequently than adults due to their tendency for bleb encapsulation and progressive anterior rotation of the intraocular portion of the tube. Furthermore, it is of utmost importance that the surgeon remains vigilant for signs of endophthalmitis in pediatric patients, because the condition tends to occur more frequently in pediatric as compared to adult patients and may require early removal of the GDD. Adults may be more prone to corneal decompensation, which also tends to occur earlier than in children.
